# Alternative polyadenylation profiles of susceptible and resistant rice (*Oryza sativa L.*) in response to bacterial leaf blight using RNA-seq

**DOI:** 10.1186/s12870-024-04839-6

**Published:** 2024-02-28

**Authors:** Shaochun Liu, Shuqi Luo, Dewei Yang, Junying Huang, Xinlei Jiang, Shangwei Yu, Junru Fu, Dahu Zhou, Xiaorong Chen, Haohua He, Haihui Fu

**Affiliations:** 1 Key Laboratory of Crop Physiology, Ecology, and Genetic Breeding, Ministry of Education, Jiangxi Agricultural University, Nanchang, 330045 China; 2 Institute of Rice, Fujian Academy of Agricultural Sciences, Fuzhou, China

**Keywords:** Alternative polyadenylation, Rice bacterial leaf blight, *Xanthomonas oryzae pv. oryzae*, RNA-seq, Rice (*Oryza sativa L.*)

## Abstract

**Background:**

Alternative polyadenylation (APA) is an important pattern of post-transcriptional regulation of genes widely existing in eukaryotes, involving plant physiological and pathological processes. However, there is a dearth of studies investigating the role of APA profile in rice leaf blight.

**Results:**

In this study, we compared the APA profile of leaf blight-susceptible varieties (CT 9737-613P-M) and resistant varieties (NSIC RC154) following bacterial blight infection. Through gene enrichment analysis, we found that the genes of two varieties typically exhibited distal poly(A) (PA) sites that play different roles in two kinds of rice, indicating differential APA regulatory mechanisms. In this process, many disease-resistance genes displayed multiple transcripts via APA. Moreover, we also found five polyadenylation factors of similar expression patterns of rice, highlighting the critical roles of these five factors in rice response to leaf blight about PA locus diversity.

**Conclusion:**

Notably, the present study provides the first dynamic changes of APA in rice in early response to biotic stresses and proposes a possible functional conjecture of APA in plant immune response, which lays the theoretical foundation for in-depth determination of the role of APA events in plant stress response and other life processes.

**Supplementary Information:**

The online version contains supplementary material available at 10.1186/s12870-024-04839-6.

## Introduction

Rice bacterial blight (BB), a bacterial disease of plants caused by Xanthomonas oryzae pv. oryzae (*Xoo.*) ranks among the top three most severe diseases of rice and has the potential to severely damage rice yields, even leading to crop failure [1]. Currently, planting varieties containing broad-spectrum resistance genes is the most cost-effective and environmentally friendly approach to control rice leaf blight [2]. Therefore, it is crucial to accumulate sufficient resources of broad-spectrum resistance (BSR) genes.

Plants are continuously exposed to various pests and diseases in a complex and variable natural environment. As a result, they have evolved innate immune regulatory mechanisms to protect themselves from invasion, including the effector-triggered immunity (ETI) and pattern-triggered immune response (PTI) via recognizing pathogen-associated molecular patterns (PAMPs) [3]. Brassinolide (BR), a novel phytohormone, is also associated with leaf morphogenesis and resistance to bacterial blight disease in rice [4].

Numerous genes related to leaf blight resistance have been discovered currently, which indicates that rice disease resistance can be enhanced by affecting gene expression levels in plant innate immune pathways. *Xa1* is an nucleotide-binding site-leucine rich repeat (NBS-LRR) type bacterial wilt disease resistance gene that exhibits specific resistance against the dominant species of Japanese bacterial wilt disease, strain 1. Notably, the increase in the *Xa1* gene expression may improve the efficiency of interaction between *Xa1* and avirulent gene avr, thereby activating the disease resistance signaling pathway mediated by *Xa1*. Therefore, *Xa1* plays a major role in the recognition process with pathogens [5]. The induction of *OsTFX1* relies on the type III effector gene pthXo6 of the bacterial wilt pathogen, and *OsTFX1* expression has been documented to elevate the susceptibility of rice to bacterial wilt disease [6]. *OsSWEET13* is a sucrose transporter gene that can serve as a susceptibility gene for the TAL effector PthXo2. Specifically, specific changes in the *OsSWEET13* promoter in japonica rice may confer potential recessive resistance to PthXo2-mediated bacterial blight [7]. *OsERF123* encodes an AP2/ERF transcription factor, a susceptibility gene to bacterial wilt disease. TalB, a transcriptional activator-like effector from the bacterial wilt pathogen, can interact with *OsERF123* and O*sTFX1,* making the host more sensitive to races X11-5A [8]. Researchers have employed a multiplex genome editing approach to generate rice lines resistant to known *Xoo* strains, which systematically disrupted the binding of *Xoo'*s transcription activator-like effector (TALE) to effector binding element (EBE) in the promoter region of the SWEET gene [9]. *OsRLR1* encodes an NLR protein with a CC-NB-LRR structure, and overexpression of O*sRLR1* can enhance the resistance of rice to bacterial blight, and the mutant exhibits favorable agrologic traits. Moreover, *OsRLR1* can enhance the transcriptional activation function of *OsWRKY19* in the nucleus, and *OsWRKY19* can bind to the promoter of *OsPR10* to activate its transcription [10]. It has been revealed that phosphorylation of TGA3 by GSK-like kinase BRASSINOSTEROID INSENSITIVE 2 (BIN2) can inhibit the expression of disease progression-related (PR) genes induced by salicylic acid (SA) and regulate plant immune response [11]. However, only a few major genes of bacterial blight in rice have been cloned, including some susceptibility genes [12].

Transcriptome profiles at the genome-wide level have been studied by omics technology, offering a valuable resource for finding genes associated with rice bacterial blight [13–17]. Recently, post-transcriptional regulation plays an important role in plant pathogen infection.

Alternative polyadenylation (APA) is an important post-transcriptional regulation in eukaryotic cells, which generates transcripts of varying lengths and participates in physiological and pathological processes [18, 19]. APA not only depends on the location of the poly (A) signaling site at the 3'end of its transcript but also involves multiple polyadenylation factors [20], e.g., the cleavage and polyadenylation specificity factor (CPSF), cleavage stimulation factor (CstF), poly (A) polymerase (PAP), cleavage factors (CF), and poly(A) binding protein (PAB) [21, 22].

More than 70% of genes in the whole rice genome have APA phenomenon with more miRNA and RNA protein binding sites [23, 24]. Notably, the knockout of *CPSF30* leads NUE signal choice from A-rich poly(A) signals to U-rich poly(A) signals in Arabidopsis, leading to enhanced tolerance to oxidative stress [23]. *HLP1* can produce biologically functional full-length FCA transcripts, which suppress the expression of the flowering repressor FLC and regulate the flowering process, and then FLC *m*ay bind to the intron of the FCA precursor mRNA and promote the use of the distal poly(A) site in the 3'UTR as well as the U-rich element [25]. *Fip1* is important for the abiotic stresses of the plant and may reduce the proximal poly(A) site [26]. In disease resistance of indica and cold-stress tolerance of japonica, genes exhibit a distinct subspecies-specific multiple poly(A) locus profile. Additionally, defense genes are regulated by APA, including R genes encoding nucleotide-binding site-leucine rich repeat (NBS-LRR) disease resistance proteins and the stripe rust resistance protein gene *Yr10*, but its poly(A) site is mainly located in the coding sequence region (CDS) [27].

Recent findings elucidate that *OsSPL4* enhances the immune response to rice blast, while *Osa-miR535* mediates the cleavage of *OsSPL4* mRNA between base pairs 10 and 11 of the predicted binding site in the 3' untranslated region, thereby suppressing the immunity to rice blast [28]. Importantly, APA-mediated lengthening or shortening of a gene can lead to the production or loss of miRNA, indicating that APA may affect miRNA binding and participate in plant pathological processes. However, the function of APA in rice bacterial blight remains unclear.

In our study, RNA-seq data for the susceptible variety CT 9737–6-1-3P-M and resistant NSIC RC15 treated with *Xoo.* P6 within 0, 12, 24, and 72 h from the NCBI database were downloaded, followed by genome-wide APA analysis utilizing bioinformatics [16]. Our results showed that these two varieties with widely differing levels of leaf blight resistance exhibited a preference for distal poly(A) profiles at the end site in response to leaf blight, but they displayed different patterns in regulating biological processes. This study elucidated the comprehensive dynamics of APA in rice in response to rice leaf blight, enriching the exploration of plant responses to biological stress at the post-transcriptional level and providing a unique perspective for scholars engaged in plant immunity research.

## Results

### Rice tends to use distal poly(A) sites in response to bacterial blight strain *Xoo. P6*

**Fig. 1:**
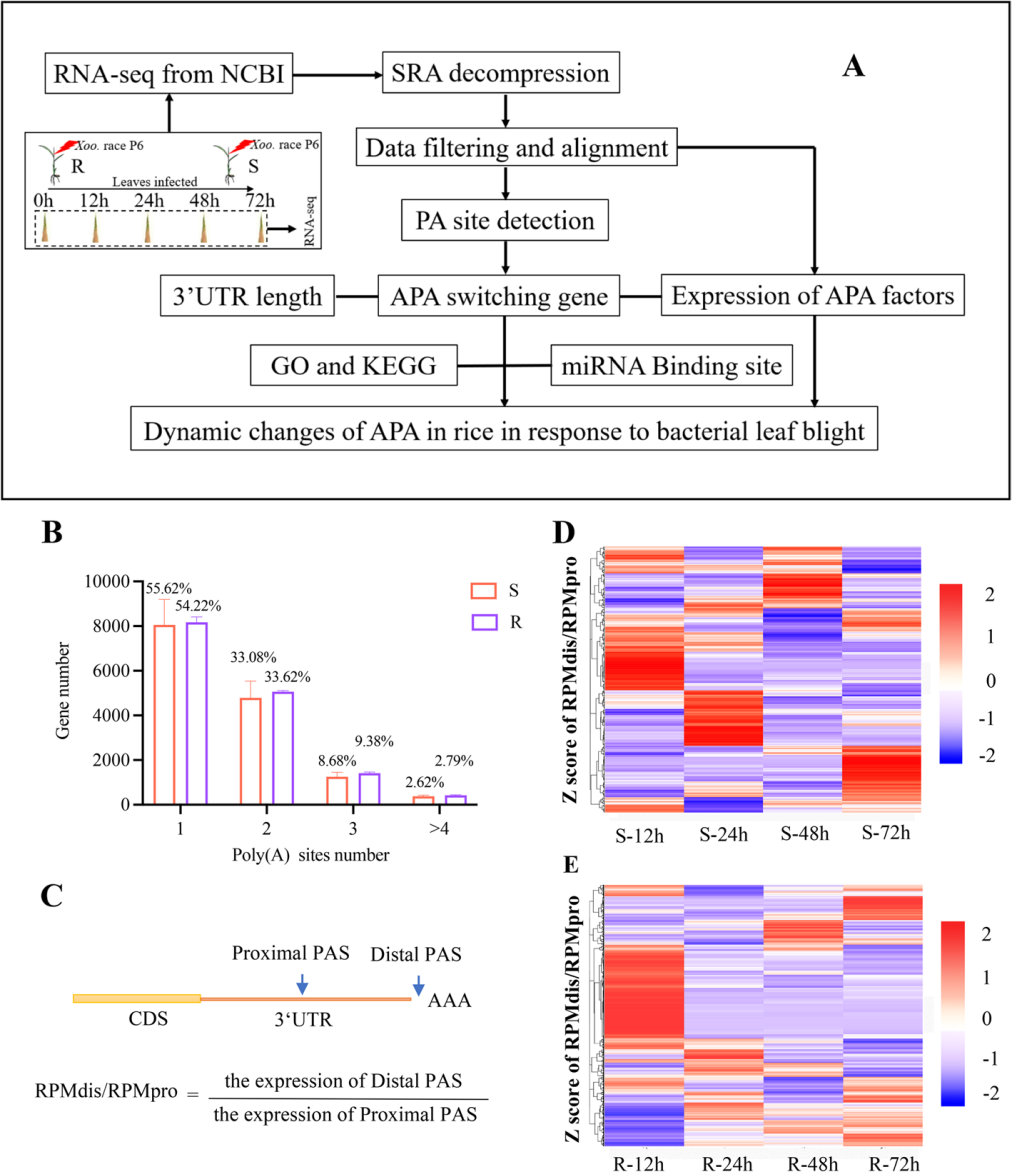
Profiles of the poly(A)(PA) sites of disease resistance and susceptibility under leaf blight stress conditions. APA: alternative polyadenylation (**A**) Flow chart of data analysis in this paper. R: resistant rice, S: susceptible rice. **B** Genes and poly(A) sites (per gene) identified in rice. **C** APA profile was calculated using the relative expression of distal PA site isoforms (RUD). **D** RUD pattern of susceptible rice. **E** RUD pattern of resistant rice

To investigate the dynamic changes and role of APA during rice response to rice leaf blight, a set of RNA-seq data was downloaded, regarding the infection of leaves of the most resistant (NSIC RC15) and susceptible lines (CT 9737–6-1-3P-M) inoculated with *Xoo.* after 0, 12, 24, 48 and 72 h (Fig. 1A). The mapped reads value was 92.63%-94.84% and 92.79–94.83% for NSIC RC154 and CT 9737–6-1-3P-M, respectively, when compared with the reference genome *O. sativa Nipponbare* (Supplementary Table 1).

**Fig. 2:**
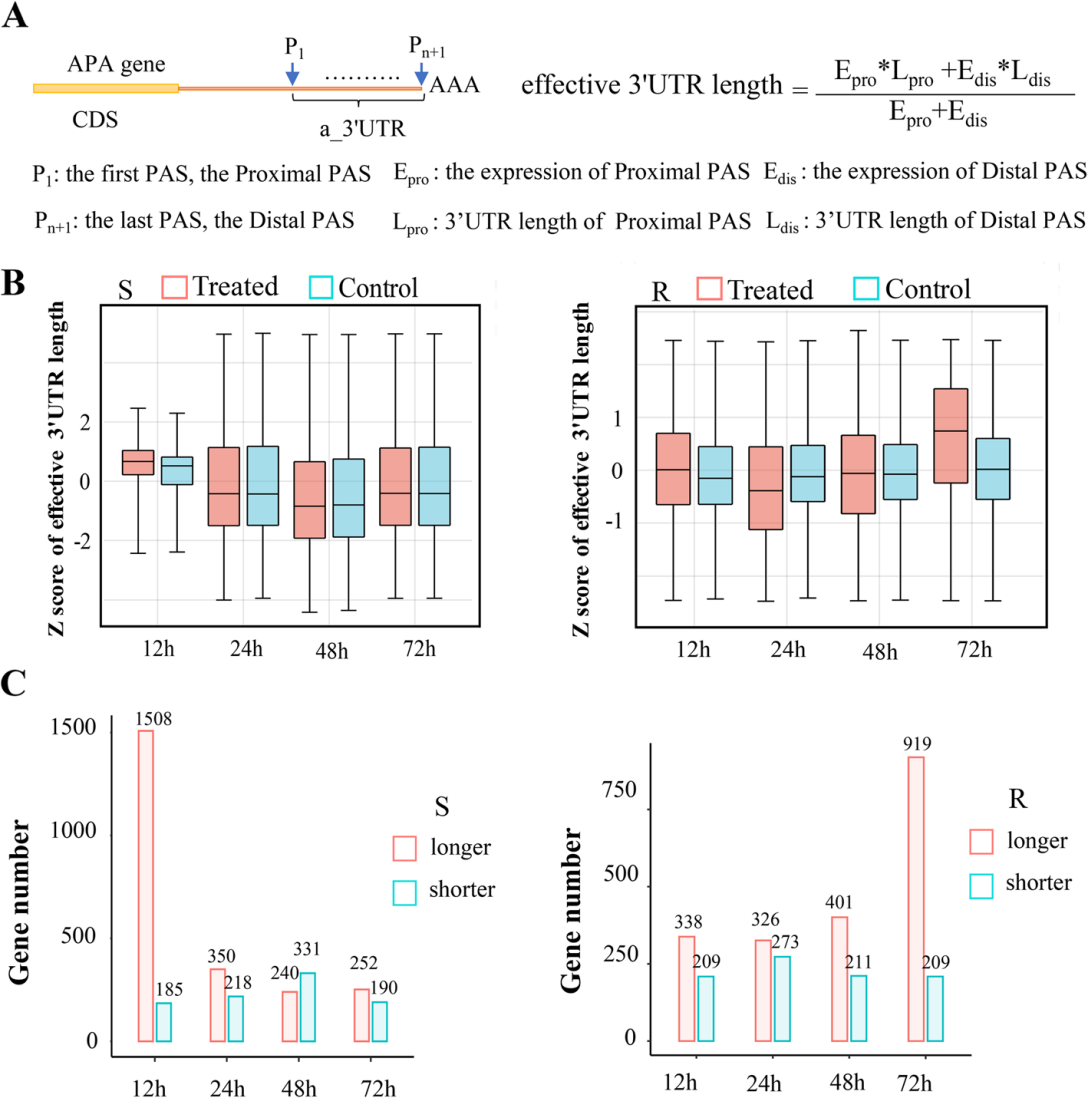
3’UTR length analysis. **A** The calculation formula for effective 3’UTR length. a_3’UTR: alternative 3’UTR. **B** Effective 3’UTR length of susceptible (S) and resistant (R) rice relative to control. **C** The number of APA genes in susceptible (S) and resistant (R) rice from APAtrap

Firstly, APAtrap was used to identify the APA locus of genes to investigate the pattern of APA changes in rice in response to rice leaf blight. APAtrap is a software that can identify multiple PA sites based on RNA-seq data [29]. Nearly half of the genes in the two rice varieties subjected to leaf blight treatment exhibited a preference for multiple PA sites (Fig. 1B), and more than 40% of the genes had 2 or more PA sites, suggesting a widespread trend in rice to utilize multiple PA sites in response to stress induced by rice leaf blight (Supplementary Material 1). Then, we exclusively focused on the ratio of distal and proximal PA sites to comprehensively examine the overall APA profile (Fig. 1C) [30]. A higher ratio suggests a greater preference for the distal end, whereas a lower ratio for the proximal end. Overall, the PA sites were dynamically utilized in the resistant rice variety NSIC RC154 and the susceptible rice variety CT 9737–6-1-3P-M (Fig. 1D, E). The selection of the PA sites affects the length of the gene 3'UTR [31]; the phenomenon of the propensity for multiple PA sites leads to large variability in the length of their 3'UTRs, so we opted to calculate the weight of the effective 3'UTR length for each gene (Fig. 2A).

It was found that susceptible rice 9737–6-1-3P-M exhibited a noticeable inclination towards an extended 3'UTR length as the infection time with rice leaf blight increased compared with the control (Fig. 2B), and this change was particularly evident at 12 h post-infection. The increase in the length of APA genes was more pronounced in the resistant rice variety NSIC RC154. The 3'UTR length of the gene was significantly longer at 12 h and 72 h compared with the control, only to become shorter at 4 h compared with the control (Fig. 2B). In terms of the number of APA genes identified (|r|> 0.15, r: Pearson correlation coefficient form APAtrap, *p*-value < 0.05), the number of longer APA genes was greater than the number of shorter genes in both rice varieties at each time point after infection with leaf blight (Fig. 2C). The trends of gene number changes at each time point were the same as the change in their effective 3’UTR length. But there was a counterexample, when infected with bacterial leaf blight in susceptible rice for 48 h, the number of shortened genes was greater than that of elongated genes. However, this trend was also reflected in the effective 3 'UTR length.

Further analysis uncovered 21 APA genes showing consistent changes in response to rice leaf blight for 72 h in the resistant rice variety NSIC RC154, of which 20 APA genes exhibited a preference towards the distal PA locus (Supplementary Table 2). These genes included three associated with abiotic stresses, namely the rice drought tolerance regulatory gene *OsSKIPa* [32], *SRWD5* [33](which are homologous to *SRWD1 i*n response to salt stress), *OsVOZ2*, a single zinc finger protein VOZ transcription factor involved in effector molecular triggering [34, 35], and *AldC-1* [36]. Similarly, the same pattern was found in the susceptible rice variety CT 9737–6 13 PM, where 23 APA genes were constantly altered in response to rice leaf blight, containing up to 19 genes that were ever lengthened (Supplementary Table 3), such as *Piz* (encoding a nucleotide-binding structural domain-containing and leucine-rich repeat sequence protein) [37, 38], *OsERF* (containing the AP2 structural domain protein), and *BZR1* [39, 40]. These results implied that some genes responding to adversity stress were regulated by APA and that APA events played important roles in the defense mechanisms of rice against rice leaf blight.

Collectively, resistant rice NSIC RC154 and rice susceptible CT 9737–6-1-3P-M have an overall longer 3'UTR in response to rice leaf blight. That is, the APA expression profiles of these two rice varieties with distinct levels of resistance demonstrate similarities, both 3'UTRs tend to become longer, and APA events may affect plant resistance-related genes.

### APA plays different roles in response to leaf blight among rice varieties

**Fig. 3:**
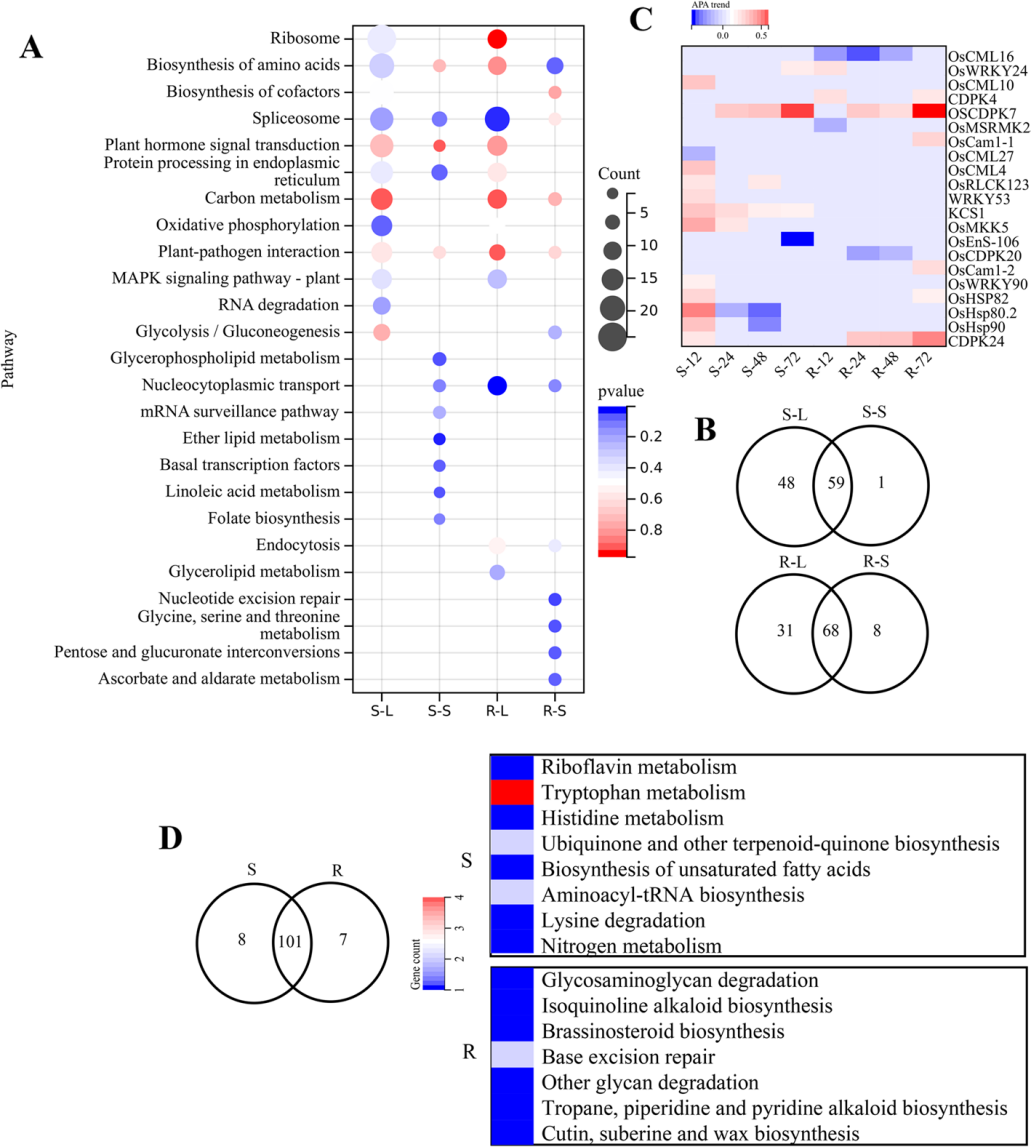
APA Genes Functional Analysis in Response to bacterial leaf blight. **A** Gene count of top 12 pathways were found in both the longer and shorter APA genes of resistant and susceptible rice with bacterial blight. **B** Overlapping pathways of long and short APA genes in resistant(R) and susceptible(S) rice infected with bacterial blight(L:longer; S:shorter). **C** APA changes of genes in the plant-pathogen interaction pathway in susceptible(S) and resistant(R) rice with bacterial blight. **D** Overlapping pathways of APA genes in resistant(R) and susceptible(S) rice infected with bacterial blight(left). Unique pathway in resistant(R) and susceptible(S) rice infected with bacterial blight(right)

Given the similarities in the APA expression profiles of resistant and susceptible rice genes, our focus shifted towards understanding the functional roles of these APA genes in response to bacterial blight and elucidating the difference in APA function in the susceptible and resistant rice. Firstly, the function of shorter and longer transcripts was explored. According to the KEGG pathway enrichment results (Fig. 3A), both longer and shorter APA genes of resistant and susceptible rice were enriched in the biosynthesis of amino acids, spliceosome, plant-pathogen interaction, and other pathways. Moreover, Venn analysis revealed 68 overlapping APA genes identified in disease-resistant rice and 59 genes in susceptible rice (Fig. 3B). Among all APA genes, 101 were also shared between susceptible and resistant rice. This also indicates that the APA changes of the genes in the three common pathways mentioned above are dynamic and universal in response to rice bacterial blight. It was further found that genes in the plant-pathogen interaction pathway directly related to plant disease resistance showed APA dynamic changes in the susceptible and resistant rice (Fig. 3C), especially calcium regulated proteins and protein kinases. A previous study has shown that long transcripts of genes are more susceptible to other targets, such as miRNA, and display reduced stability and translation efficiency than short transcripts [41]. Therefore, APA may participate in plant response to bacterial blight by regulating these important genes involved in the interaction between pathogens and plants.

In the present study, genes at the distal end of the PA locus tendency were associated with phytohormone regulation in response to rice leaf blight in the susceptible rice variety CT 9737–6-1-3P-M (Supplementary Table 4). *OsRTH1* [42], a regulator of ethylene response in rice, along with ethylene receptor *ETR3* [43, 44], ethylene signaling regulator *OsEIL3* [45, 46], and abscisic acid (ABA) receptor *OsPYL10* [47, 48] all exhibited a preference for distal PA sites and demonstrated an up-regulation in expression, including mitogen-activated protein kinase (MAPK) signaling cascade associated Mitogen-activated protein Kinase Kinase 5 (*OsMKK5*). These APA genes have a pivotal role in recognizing signals and activating plant defense mechanisms, thus suggesting their extensive involvement in the defense response to *Xoo.* infection in rice.

In addition, there were eight unique genes in resistant rice, which were involved in brassinosteroid biosynthesis, cutin, suberine, and wax biosynthesis (Fig. 3D). In recent years, studies on brassinosteroid have revealed their involvement not only in leaf angle formation but also in their interaction with transcription factors for plant disease resistance [49]. Additionally, cutin, suberine, and wax serve as the first lines of defense against pathogenic bacteria in rice [50]. Seven APA genes were identified to be associated with riboflavin metabolism, histidine metabolism, and nitrogen metabolism. These illustrated that APA events in resistant rice NSIC RC154 and susceptible CT 97376–1-3P-M were involved in completely different biological functions in response to rice leaf blight, which further revealed the significant role of the unnoticed polyadenylation process in response to bacterial leaf blight.

### Significant changes in APA genes associated with leaf blight resistance

It has been documented that APA can affect the expression of genes by altering the target site on the variable transcript [51]. The above analysis showed that all APA genes tended to utilize distal PA sites in response to rice leaf blight. Then, a comparative analysis was performed based on the 3'UTR changes of APA genes and their gene expression patterns.

**Fig. 4:**
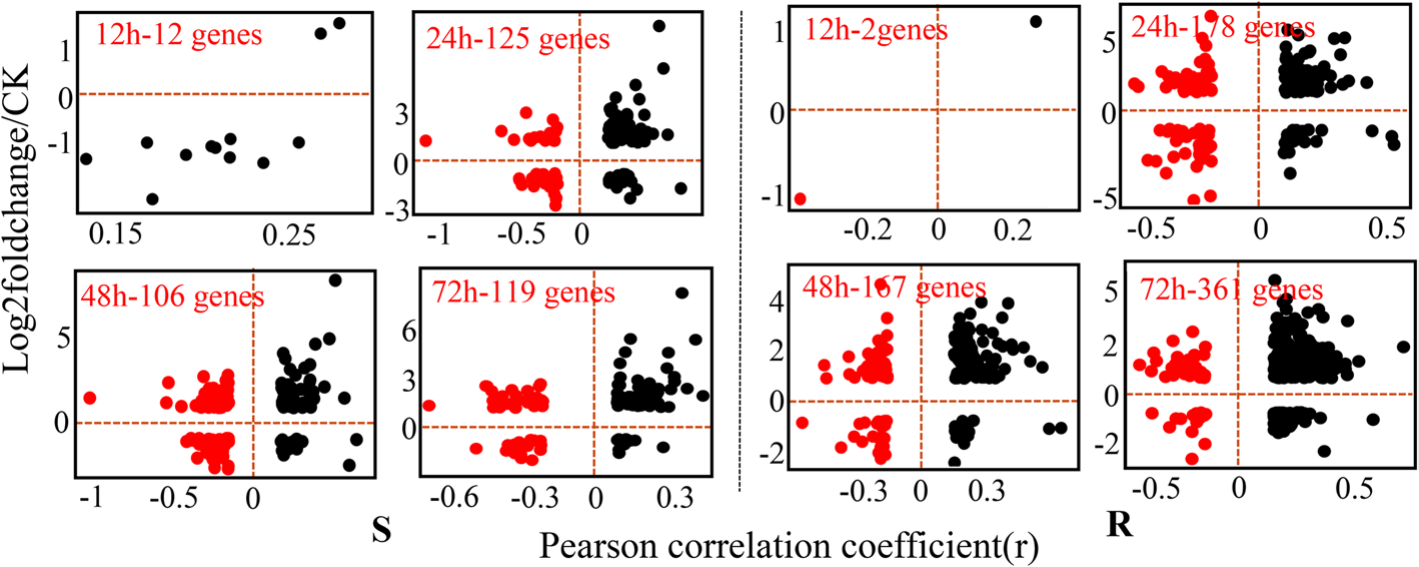
Dynamic distribution of DE-APA genes after rice bacterial blight with infection (DE-APA: APA genes with significant changes in expression relative to controls). The susceptible cultivar is shown on the left, and the resistant cultivar is shown on the right. (r: the tendency of 3'UTR variation; r > 0: the 3'UTR of a gene tends to be longer, and a higher value indicates a greater tendency to be longer)

According to the above studies, many differentially expressed APA genes (DE-APA, |log2foldchange|> 1, *p*-adjust < 0.05) associated with rice disease resistance were found in this analysis (Supplementary Table 5). The total number of DE-APA genes in resistant rice was higher than that in susceptible rice. Unlike the APA event, the number of DE-APA genes in both rice cultivars was the lowest at 12 h after infection, and DE-APA reached the peak at 72 h in susceptible and resistant rice (Fig. 4).

We found that genes exhibiting a negative correlation between APA changes and differential expression may be more susceptible to APA, so we conducted an investigation into the functions of DE-APA genes that display a negative correlation between them. DE-APA was found to be associated with glycerophospholipid metabolism and Phenylpropanoid biosynthesis in the susceptible (Table 1).

**Table 1:** Enrichment pathways of DE-APA genes whose expression levels were negatively correlated with APA trend in susceptible rice after bacterial blight

	Description	*p*value	geneID	Count
s	Photosynthesis—antenna proteins	0.033237947	*Os01g0600900*	1
	Ether lipid metabolism	0.037595793	*Os02g0119800*	1
	Phenylalanine metabolism	0.052713201	*Os02g0626100*	1
	Autophagy—other	0.073948962	*Os04g0682000*	1
	Phenylalanine, tyrosine and tryptophan biosynthesis	0.082325908	*Os09g0565700*	1
	Glycerophospholipid metabolism	0.148944159	*Os02g0119800*	1
	Ubiquitin mediated proteolysis	0.181580766	*Os01g0104600*	1
	Phenylpropanoid biosynthesis	0.274714733	*Os02g0626100*	1
	Biosynthesis of amino acids	0.338344331	*Os09g0565700*	1
	Ribosome	0.430841955	*Os05g0528900*	1

In resistant rice, most APA genes involved splicesome, plant-pathogen interaction, and the phosphatidylinositol signaling system, such as disease resistance pathways (Table 2). This further indicates that APA plays distinct roles in the response of resistant and susceptible rice to bacterial blight and regulates the resistance of resistant rice to bacterial blight. Moreover, many resistance genes exhibited significant changes in their expression levels, such as the rice blast resistance gene Piz (*Os06g0286700*), the downstream signaling molecule *BZR1* (*Os07g0580500*) for oleuropein lactone signaling, *SLR1* (*Os03g0707600*) whose mutant was resistant to Xoo. race *PXO99,* and magnaporthe oryzae race *VT7* [52], *Pia* (*Os11g0225100*), which was associated with leaf blight resistance in rice [53]. Accordingly, we infer that APA events may regulate disease-resistance genes involved in the immune process of rice in response to *Xoo*. invasion, highlighting the critical role of APA in the response of rice to pathogens.

**Table 2:** Enrichment pathways of DE-APA genes whose expression levels were negatively correlated with APA trend in resistant rice after bacterial blight

	Description	*p*value	geneID	Count
R	Inositol phosphate metabolism	0.063248525	*Os03g0726200/Os10g0103800*	3
	Phosphatidylinositol signaling system	0.068656835	*Os03g0726200/Os10g0103800*	3
	Lysine biosynthesis	0.103093931	*Os04g0254000*	3
	Arachidonic acid metabolism	0.103093931	*Os03g0819100*	2
	Linoleic acid metabolism	0.120694688	*Os03g0179900*	2
	Nucleocytoplasmic transport	0.143164633	*Os01g0383900/Os03g0110400*	2
	Ether lipid metabolism	0.154895276	*Os02g0119800*	2
	Spliceosome	0.164039354	*Os07g0633200/Os02g0266100/Os03g0110400*	2
	Folate biosynthesis	0.187802196	*Os02g0140300*	1
	RNA polymerase	0.242418476	*Os07g0615800*	1
	Biosynthesis of amino acids	0.256379679	*Os09g0565700/Os04g0254000/Os05g0429000*	1
	Terpenoid backbone biosynthesis	0.334537802	*Os06g0167400*	1
	Nucleotide excision repair	0.347707494	*Os02g0179300*	1
	Glycine, serine and threonine metabolism	0.379533741	*Os05g0429000*	1
	Ribosome	0.428550236	*Os02g0198900/Os01g0304000/Os03g0751400*	1
	Glyoxylate and dicarboxylate metabolism	0.444332092	*Os05g0429000*	1
	Proteasome	0.444332092	*Os02g0182500*	1
	Plant-pathogen interaction	0.451293367	*Os07g0568600/Os03g0128700*	1
	Glycerophospholipid metabolism	0.507524189	*Os02g0119800*	1
	Biosynthesis of nucleotide sugars	0.512463058	*Os03g0832600*	1
	Cysteine and methionine metabolism	0.576755287	*Os02g0771600*	1
	Amino sugar and nucleotide sugar metabolism	0.706562415	*Os03g0832600*	1
	MAPK signaling pathway—plant	0.71250573	*Os03g0610900*	1
	Plant hormone signal transduction	0.873121231	*Os03g0610900*	1

### Ninety percent of APA genes have miRNA targets in the variable 3'UTR region

**Fig. 5:**
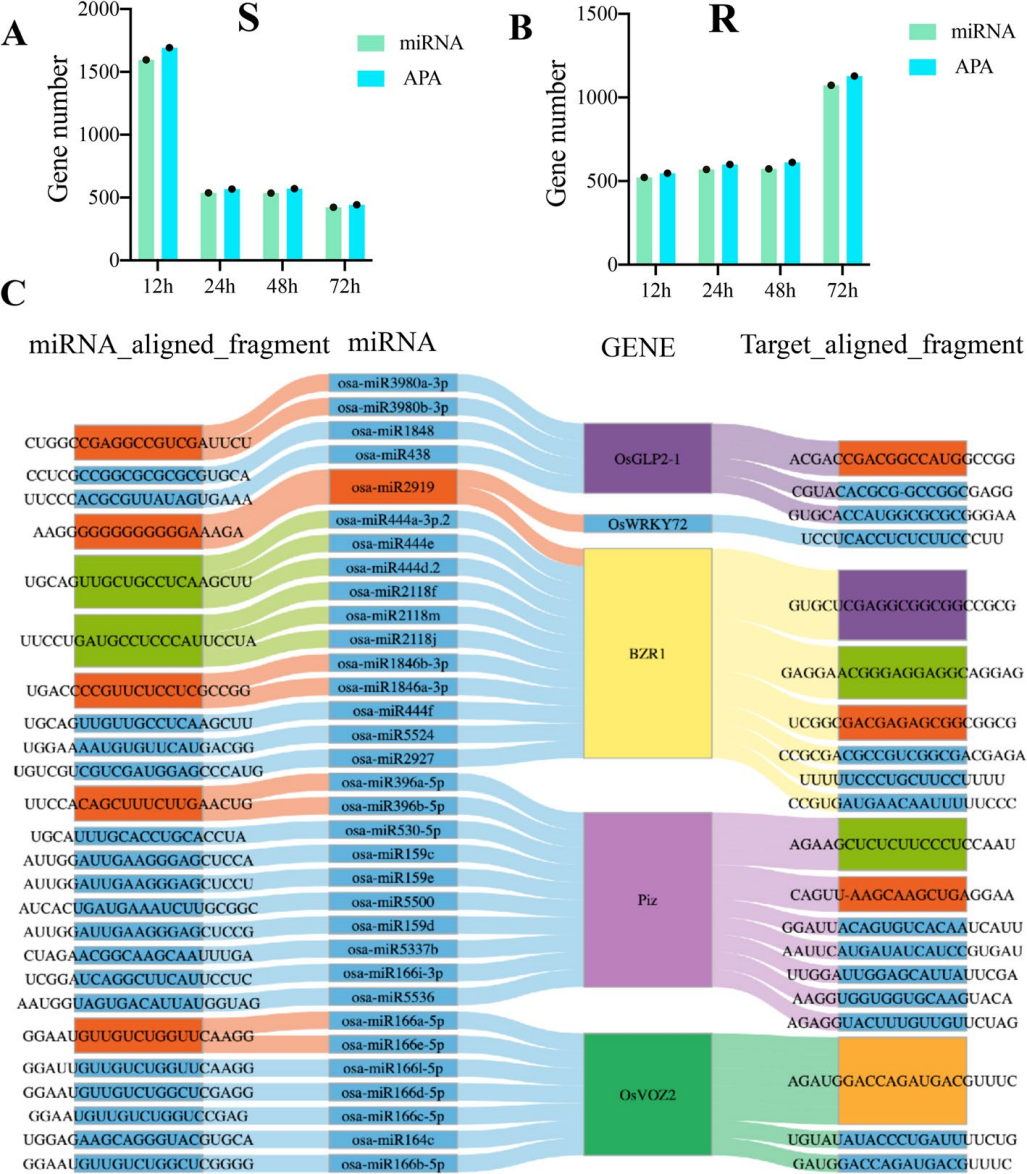
MiRNA targets APA genes with alternative 3’UTR region (a_3’UTR) in response to bacterial blight. **A** APA genes containing miRNA target sites in susceptible rice. **B** APA genes containing miRNA target sites in resistant rice. **C** MiRNA and their binding sequences in a_3’UTR of candidate genes

Notably, miRNAs are small RNAs (1 to 22 nt), and genes may introduce binding sites for miRNAs when selecting distal PA sites, leading to post-transcriptional gene silencing (PTGS), which in turn affects both transcript stability and translation efficiency [41, 51]. Numerous rice miRNAs have been identified as fine-tuners regulating agronomic traits and immunity. To further explore the effect of 3ʹUTR-APA on gene expression, we predicted miRNA target sites on the variable 3'UTR of APA genes in all samples. Overall, miRNA binding sites were present in the variable 3ʹUTR region of over 90% of APA genes (Fig. 5A, B). This indicates that APA has a direct impact on the length of transcripts and can introduce or evade miRNA targets, ultimately influencing gene expression. Genes with high frequency in our study, *Piz* (*Os06g0286700*) and *OsBZR1* (*Os07g0580500*) in susceptible rice, *OsVOZ2* (*Os05g0515700)*, *OsGLP2-1* (*Os02g0532500*), and *OsWRKY72* (*Os11g0490900*) had miRNA binding in the variable 3' terminal UTR (Fig. 5C), and these APA genes had up-regulated expression and longer transcripts compared with the control (0 h). The mentioned genes have been linked to rice leaf blight, with the exception of the rice blast resistance gene *Piz*. APA events have been reported to alter the transcript length of R genes. Based on the aforementioned information, polyadenylation events under bacterial *Xoo*. stress may be indirectly involved in rice immune activity by altering the transcript length of rice genes and affecting miRNA targets and gene expression levels.

### Polyadenylation factors show a similar downward expression trend under the bacterial blight

To investigate the potential mechanisms that drive the preference for poly(A) site use in rice response to leaf blight disease as trans-acting elements, the expression dynamics of core cleavage and polyadenylation factors have been noted to influence APA profiles [23, 54, 55]. Therefore, the relevant core polyadenylation factor expression from the transcriptome data (Supplementary Table 6) was extracted, followed by trend analysis.

**Fig. 6:**
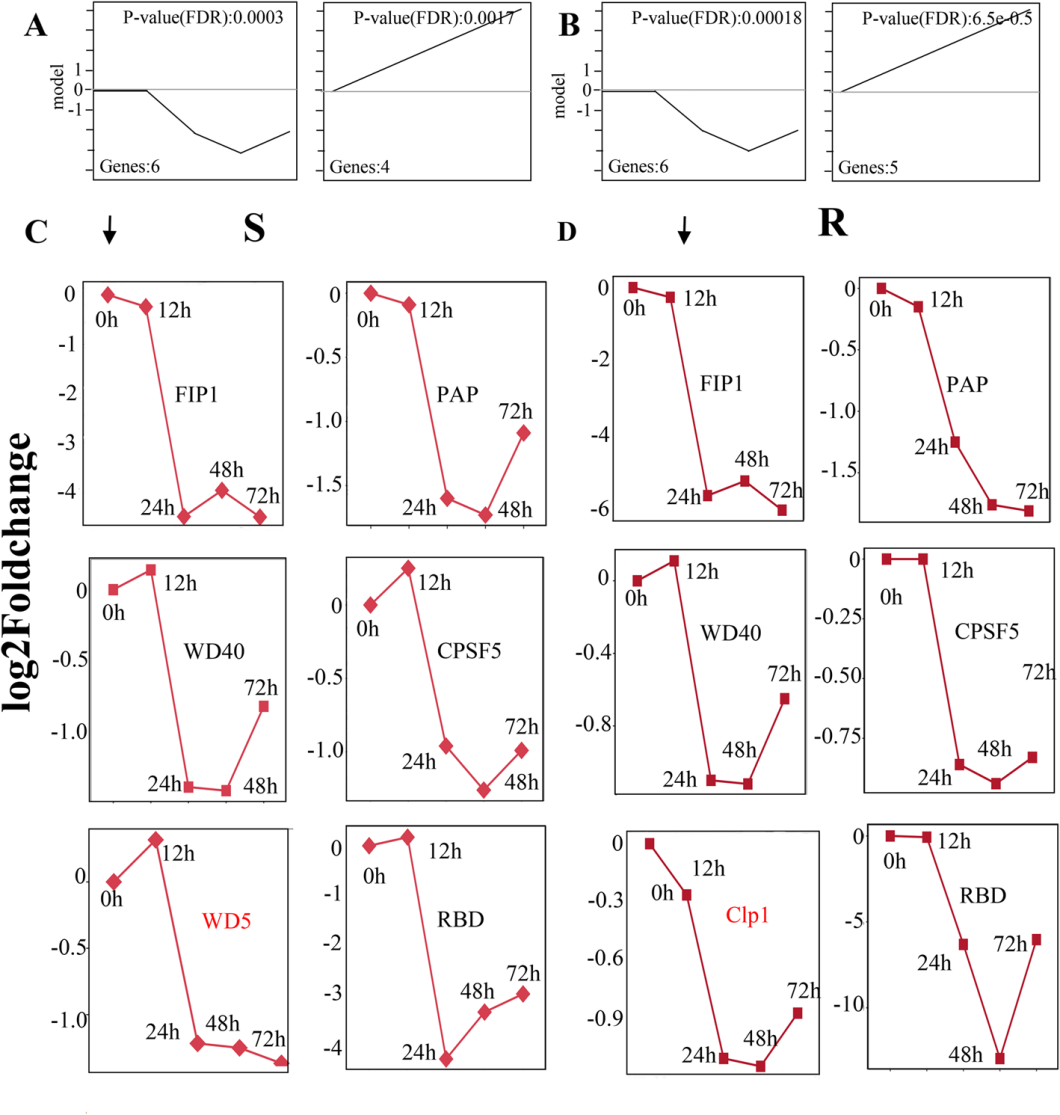
Expression patterns of polyadenylation factors in resistant cultivars infected with bacterial blight. **A** Trend patterns of polyadenylation factors in resistant cultivars infected with bacterial blight. **B** Trend patterns of Polyadenylation factors in susceptible cultivars infected with bacterial blight. **C** The curve of the expression of downregulated polyadenylation factors in resistant cultivars infected with bacterial blight. **D** The curve of the expression of downregulated polyadenylation factors in susceptible cultivars infected with bacterial blight

It was found that these polyadenylation factors underwent dramatic dynamic changes in both varieties of rice. Based on the expression changes in the factors, the relevant core polyadenylation factors were classified into two major categories utilizing the stem software, up-regulation and down-regulation (Fig. 6A, B). It was shown that the changes in the core factors were more pronounced in the down-regulation mode than in the up-regulation mode, and such a phenomenon was observed in both rice varieties in response to rice leaf blight. Six factors were down-regulated overall in both varieties. There were 5 genes, and 4 genes were upregulated in disease-susceptible and disease-resistant rice. Among the down-regulation patterns, we found stress-related factors, such as* Fip1*, that were shown to affect global APA expression profiles under abiotic stress in Arabidopsis [26, 56–58], *WD40* repeat sequence mutants favor short 3'UTRs, and the PPLPP structural domain lacked a preference for long transcripts [59]. Additionally, an in-depth analysis of the down-regulation pattern factor change trends (Fig. 6C, D) found the same change profile in the first 24 h post-infection. It is very interesting that the first 12 h post-infection showed little change and a dramatic down-regulation trend in the 12–24 h time. For example, *WD5* was up-regulated 0.19-fold after 12 h and down-regulated 0.73-fold after 24 h in resistant rice leaf blight treatment; *RBD* was up-regulated 0.17-fold after 12 h and down-regulated 4.23-fold after 24 h; *Fip1* was down-regulated 0.24-fold after 12 h, and down-regulated 4.48-fold after 24 h. More interestingly, five of the six factors exhibited almost identical expression trends in susceptible and resistant rice in response to rice leaf blight, namely *WD40* (*Os03g0754900*), *CPSF5* (*Os04g0683100*), *RBD* (*Os07g0602600)*, *Fip1 (Os03g0308900),* and *PAP (Os07g0688500)*. Moreover, the leaf spot gene *spl5* was identified in an up-regulation pattern of both species, negatively regulating cell death and resistance response by modulating the splicing of plant RNA [60, 61]. *Spl5* was slightly up-regulated in both species, 0.2-fold after 12 h, 0.22-fold after 24 h, 0.38-fold at 48 h, and 0.5-fold at 72 h. Taken together, these factors suggest a prevalent role in plants in response to biotic stress, in contribution to the propensity of distal PA sites, and in the plant to exert innate immunity during rice response to leaf blight.

### Rice cultivars CBB23 and JG30 exhibited a longer APA expression profile with bacterial blight infection

To verify the universality of our findings, APA expression profiles of CBB23 (harboring *Xa23*) and JG30 (without *Xa23*) during bacterial blight infection were studied [62]. Also used APAtrap computing the APA trend of two near-isogenic lines (NILs) inoculated with *Xoo.* after 0, 12, 24, 36 and 48 h (| r |> 0.15, *p*-value < 0.05) (Supplementary Material 2). In general, they also showed a trend of longer APA, and these changes were like previous studies (Supplementary Fig. S1A, B). For example, in resistant rice CBB23, longer APA genes were always more than shorter APA genes, which was like the change in APA number of NSIC RC154 (the resistant) (Supplementary Fig. S1A). Even more interestingly, two groups of rice in susceptible and resistant respectively had 1554 and 1285 APA genes overlapping, they are both more than 50% of NSIC RC154 (the resistant) and CT 9737–6-1-3P-M (the susceptible) (Supplementary Fig. S1D). It shows that the experiment method is feasible. Then, to further explore the function of longer and shorter APA genes in CBB23 and JG30, KEGG enrichment analysis showed that both the longer and shorter were enriched in biosynthesis of amino acids, spliceosome, plant-pathogen interaction, and other pathways (Supplementary Fig. S1C). Moreover, most of the pathways enriched for APA genes in CBB23 (with or without *Xa23*) and JG30 (without *Xa23*) were identical (Supplementary Fig. S1E). The above enrichment analysis results were all consistent with the previous results. And the PA site of genes in the plant pathway interaction pathway also undergoes dynamic changes, as previously studied, especially calcium regulated proteins and protein kinases. So, we can conclude that the analysis results of this study are reliable.

## Discussion

### Variation of 3′ UTR length via APA is an important regulation of genes in response to leaf blight in rice

APA occurring in different regions of genes leads to varying mRNA stability, and nonsense-mediated mRNA decay is down-regulated under plant stress, thus contributing to the accumulation of non-classical mRNAs that ensure defense-responsive gene expression [63]. This study found that long 3′ UTRs were used in two types of differentially resistant rice in response to rice leaf blight within the APA events in 3′UTRs, and their 3′UTRs demonstrated significant up-regulation. It was shown that the regulation of genes associated with the occurrence of rice bacterial blight is similar. However, the genes of variable 3′UTRs between resistant and susceptible rice were enriched in different pathways, indicating that susceptible and resistant rice responded to bacterial blight through genes in different pathways.

Interestingly, a majority of the 3′UTR variable-length genes in susceptible rice were associated with response stimuli, whereas most of the spliceosomes were observed in resistant rice. These results suggested that numerous stress-related genes and transcription factor expression were regulated by APA, and the regulation of APA events differed in response to leaf blight stress in resistant rice NSIC RC154 and susceptible 9737–6-1-3P-M. Overall, the above results revealed the crucial role of APA regulation in response to rice leaf blight disease.

### APA regulation participates in the innate immune pathway of plants

We found that many known stress tolerance genes were regulated by APA events, thereby serving a functional role in plant response to external environmental stimuli [64]. A previous study has suggested that APA events are involved in the MAPK signaling pathway and the plant hormone signal transduction-related pathway in response to six biotic and abiotic stresses in rice [19], where APA genes in three rice species subjected to bacterial leaf blight are inclined to distal PA site. Similarly, our RACE-PCR experiments demonstrated that variable shearing of two NBS-LRR-encoding genes in Arabidopsis occurred in the 5' or 3' UTR region. Besides, eight NBS-LRR-encoding R genes possessing multiple transcripts have also been reported earlier [65]. In our study, the NBS-LRR structural broad-spectrum rice blast resistance gene *Piz* was detected to be also regulated by APA in susceptible rice with a fourfold change in expression, and its 3' UTR region was predicted to be a miRNA target. Moreover, *OsSWEET14* allele *xa41(t)* [66], resistant to bacterial blight, is longer in susceptible rice, with a more than twofold change in its expression.

**Fig. 7:**
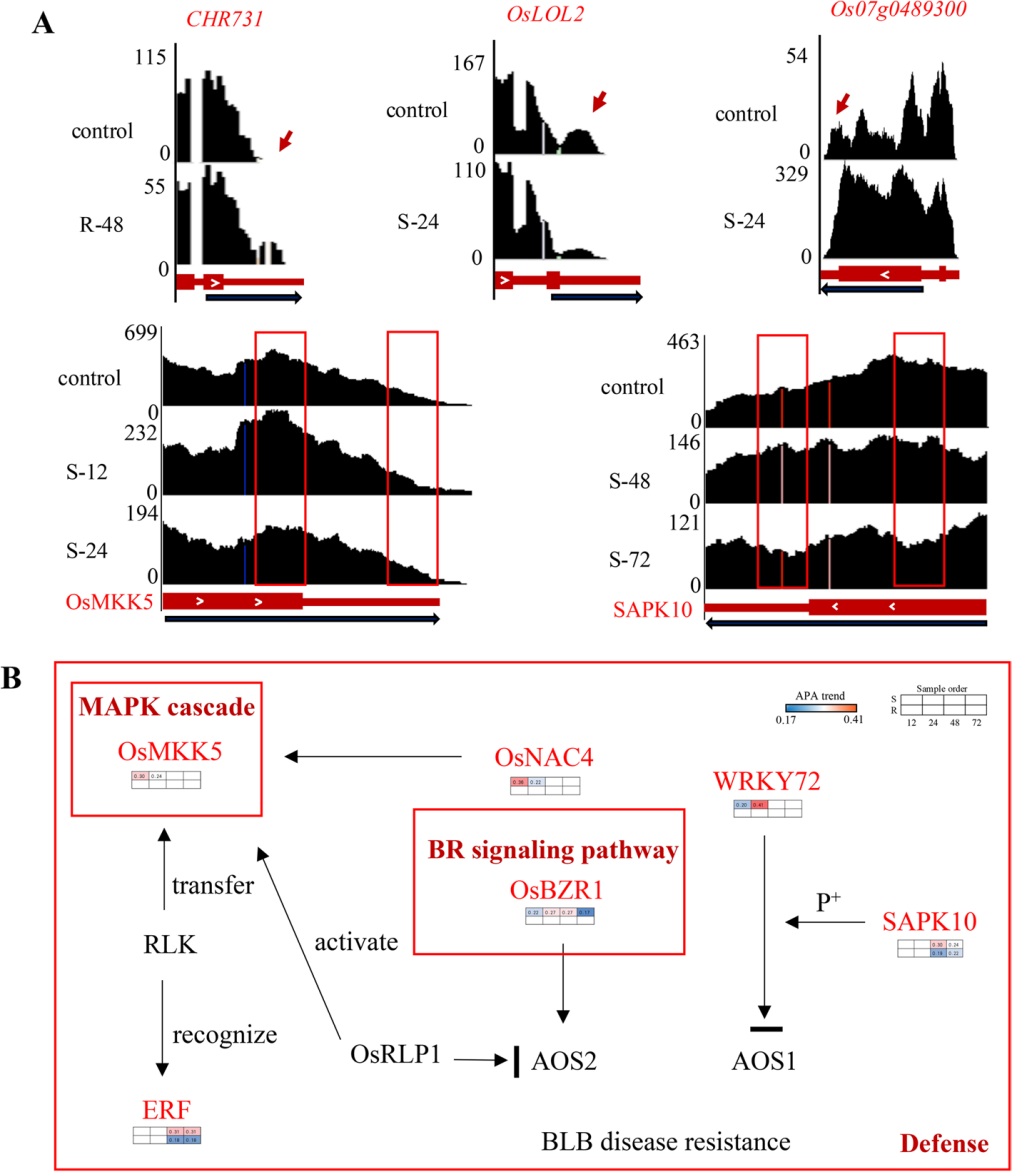
APA dynamics of genes responsible for bacterial blight (BB) stress responses in rice. **A** Integrative Genomics Viewer (IGV) showing the poly(A) sites of APA genes. Control: no infection was used as control. The red arrow and the red square indicate the PA sites. The black arrow represents a_3’UTR. **B** Regulatory networks involved in APA genes in response to bacterial blight. The APA gene is shown in red. The heatmaps show the length-change tendency of the 3′UTRs of genes. Eight squares represent eight samples, the first is susceptible rice, the second is resistant rice, and columns 1 to 4 represent 12 h, 24 h, 48 h, and 72 h after bacterial blight infection. Small red boxes refer to pathways and reactions involved by genes. p^+^ represent phosphorylation

This study also found that APA events may negatively regulate resistance to *Xoo.* In our study, *BZR1* was consistently longer in the 3 'UTR within 72 h of infection susceptible rice. It has been shown that *BZR1 i*s an important link between the BR signaling pathway and PTI response interconnected with negative regulation of resistance to *Xoo* [67]. *OsMKK5* favored long transcripts within 24 h of infection in susceptible rice, and its expression was also down-regulated (Fig. 7A). Activation of MAPK is an important response in PTI immunity, and the *OsMKK5* gene contains two alternatively spliced isoforms that form a MAPK signaling cascade with *OsMPK3/OsMPK6* to regulate PAMP signaling (pathogenic fungal pattern receptors), thus mobilizing defense responses [67]. It is shown that APA events may affect key factors in the immune response to pathogenic fungi in rice, specifically those involved in the BR signaling pathway and MAPK signaling cascade response. The previous study has reported the involvement of the *OsMAPKK4-OsMAPK6* cascade response in the regulation of BR signaling in rice [68]. In our study, these two signaling pathways were simultaneously regulated by APA at the post-transcriptional level (Fig. 7B). Although APA events are involved in broad-spectrum defense responses against leaf blight and rice blast such as the R gene, the BR signaling pathway, and the MAPK signaling pathway were identified for the first time. However, the regulatory network involved appears to be highly complex, as the relationship between the 3'UTR length and expression changes has yet to be explored.

### Polyadenylation factors are downregulated in bacterial blight P6 stimulation

Pre-mRNA processing requires the involvement of polyadenylation factor complexes. These complexes recognize multiple cis-acting elements-polyadenylation signals that polymerize into protein complexes, direct other machinery such as cleavage factors to form mRNA 3' ends, and even coordinate with other RNA processing processes to regulate gene expression [69]. *CPR5* is an RNA binding protein belonging to the SR family, and Wang Shui's team found through genetic screening that *CPR5* forms a complex with *PRL1* (a core member of the RNA splicing factor NTC complex) and *Fip1* (a core member of the RNA poly(A) plus tailing factor CPSF complex), and this complex co-localizes at the nuclear spot of the RNA processing organelle. Besides, activating plant immunity downstream of *CPR5* regulates ETI [70]. Previous studies have found that *FY* can influence single nucleotide usage and 3' UTR length preference. *WD40* repeat mutations in *FY* may directly affect AAUAAA signaling recognition [59]. In addition, polyadenylation factors can enhance plant atypical poly (A) site utilization in response to an excess of salt or cadmium, and *Fip1* has been detected in rice [71]. Using C2C12 cells, the knockdown of Fip1 lengthens the 3’UTR of nearly a thousand genes [72]. Our study revealed consistent down-regulation of five polyadenylation factors in rice infections with *Xoo.* race P6 for 72 h compared to pre-infection (Fig. 6). Apparently, the downregulation of these splicing factors may lead to lengthening the 3 'UTR of rice genes in response to bacterial blight.

In Arabidopsis, the RNA variable poly(A) plus tail regulates plant immunity, particularly through the *CPR5* signaling pathway [70]. We speculate that *Fip1*, a core member of the RNA poly(A) plus tail factor CPSF complex, binds to *CPR5* upon stimulation by *Xoo.* race P6 to activate a downstream complex innate immune network that links RNA processing and plant immunity. *WD40*, on the other hand, may affect the recognition of poly(A) signaling sequences (AAUAAA). However, further studies are needed to investigate the remaining three core factors.

## Conclusion

In summary, the selection of poly (A) site in rice undergoes substantial genome-wide changes in response to bacterial leaf blight. Such changes underscore the significance of APA in plant gene expression in response to biotic stresses and the resulting large number of unknown transcripts, including some defense genes. Although the precise nature of these transcripts is largely uncertain at present, they likely play a crucial role in the complex functional network of plants. However, the gene networks regulated by APA seem to differ in plants harboring diverse genetic resources.

## Materials and methods

### Data sources

To investigate the dynamics of overall polyadenylation in rice in response to rice leaf blight, transcriptome data from two varieties with significant differences in the field identification of leaf blight were downloaded from the NCBI database, and the SRA number is SRP261005 [16]. The resistant rice varieties (NSIC RC154) and susceptible rice varieties (CT 9737–6-1-3P-M) were injected with the most potent strain Xoo. race P6 utilizing the leaf-clipping method and then incubated under greenhouse conditions (Fig. 1A). Three replicates of 0, 12, 24, 48, and 72 hpi leaves were collected for each variety, and non-inoculate fresh leaves of seedlings at 12 h were used as controls. The data SRA numbers for CB230 and JG30 is SRP132257 [62].

### Data analysis

Firstly, the downloaded RNA-seq data files were decompressed, and then HISAT2 (2.1.0) with the reference genome and gene model annotations of *O. sativa Nipponbare* IRGSP-1.0 from ensemble plant (https://ftp.ebi.ac.uk/ensemblgenomes/pub/release-55/ plants/fasta/oryza_sativa/dna/) was used for comparison to generate SAM format files [73]. Afterward, it was converted to binary BAM files using samtools, and read matrices were obtained using subread-featurecounts (version 1.5.3). DESeq2 and ggplot2 were performed for inter-sample principal component and cluster analysis based on gene read counts. The R package DESeq2 was selected to calculate differentially expressed genes for each sample (*p*-value < 0.05, |log2foldchange|> 1).

### Identification of poly(A) sites

The comparison result files were converted into bedgraph format with the help of Samtools [74] and bedtools [75] and then imported into APAtrap to calculate PA sites [29]. Differential APA genes were screened according to *p*-value < 0. 05, and |r|> 0.15. RNA-seq coverage of genes was visualized by Integrative Genomics Viewer (IGV) v2.8.3 [76].

### 3′UTR length analysis

To compare gene usage of PA sites in different samples, effective 3'UTR [30] (Fig. 2A) and relative expression using distal PA site isoforms (RUD) (Fig. 1C) [77] were used.

### Functional enrichment analysis

According to the default parameters, Gene Ontology (GO) enrichment analysis was performed using the online software PlantRegMap (http://plantregmap.gao-lab.org/index.php) (default parameters), and Kyoto Encyclopedia of Genes and Genomes Analysis (KEGG) enrichment analysis was performed using the online website kobas (http://kobas.cbi.pku.edu.cn) (*p* value < 0.05). The Venn diagram was pictured by the online tool Venny 2.1.0 (http://bioinfogp.cnb.csic.es/tools/venny/).

### miRNA target prediction of APA genes

The presence of complementary sequence miRNAs in the gene prediction sequence region was identified as miRNA target genes. The study only calculated miRNA binding on the alternative 3'UTR (a_3'UTR) from the nearest segment PA site to the most distal PA site, employing psRNAtarget for prediction with default parameters (https://www.zhaolab.org/ psRNATarget/) [78].

### Analysis of polyadenylation factor expression patterns

To investigate the factors influencing the selection of rice PA sites in response to leaf blight, stem analysis was performed based on reads standardized by polyadenylation factors using the OuYiCloud platform (https://cloud.oebiotech.cn/task/detail/stem).

### Supplementary Information

**Supplementary Material 1.**

**Supplementary Material 2.**

**Supplementary Material 3.**

**Supplementary Material 4.**

**Supplementary Material 5.**

**Supplementary Material 6.**

**Supplementary Material 7.**

**Supplementary Material 8.**

**Supplementary Material 9.**

## Data Availability

The data were downloaded from the National Center for Biotechnology (NCBI) Sequence Read Archive (SRA) (SRP261005 and SRP132257).

## Abbreviations

APA: Alternative polyadenylation
PA: Poly(A)
BB: Rice bacterial blight
BSR: Broad-spectrum resistance
ETI: The effector-triggered immunity
PTI: Pattern-triggered immune response
PAMPs: Pathogen-associated molecular patterns
NBS-LRR: Nucleotide-binding site-leucine rich repeat
GO: Gene Ontology enrichment analysis
KEGG: Kyoto Encyclopedia of Genes and Genomes Analysis
IGV: Integrative Genomics Viewer
a_3'UTR: Alternative 3'UTR
RUD: The relative expression of distal PA site isoforms
