# Supplementary figures and images for: Alternative polyadenylation profiles of susceptible and resistant rice (Oryza sativa L.) in response to bacterial leaf blight using RNA-seq
Source: BMC Plant Biol. 2024 Feb 28;24:145. doi: 10.1186/s12870-024-04839-6 (PMC10900630; doi:10.1186/s12870-024-04839-6)

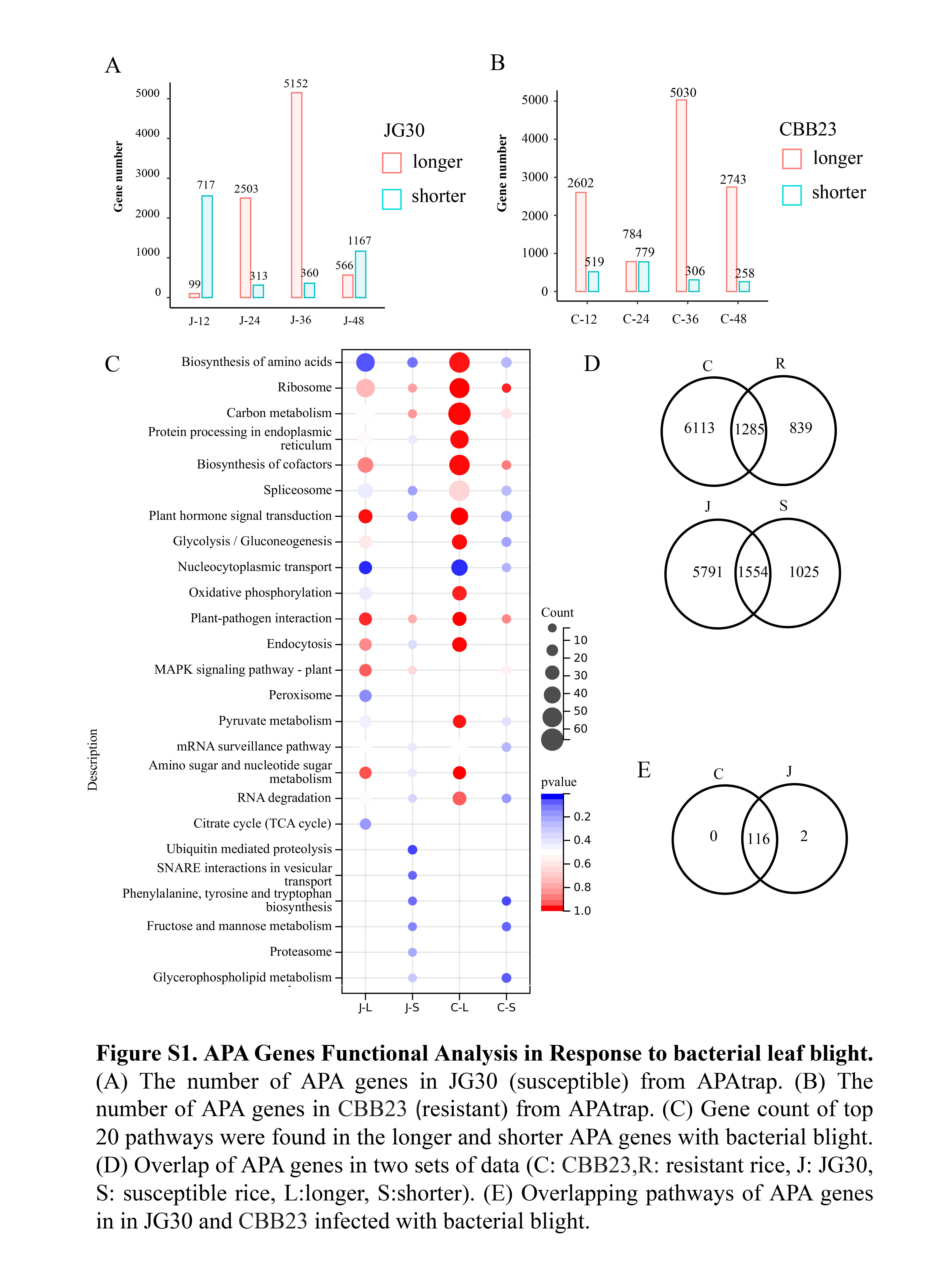

Supplement: Supplementary file 9 — Supplementary Material 9. [file 12870_2024_4839_MOESM9_ESM.tif]
